# Taxanes in the Treatment of Advanced Gastric Cancer

**DOI:** 10.3390/molecules21050651

**Published:** 2016-05-17

**Authors:** Byung Woog Kang, Oh-Kyoung Kwon, Ho Young Chung, Wansik Yu, Jong Gwang Kim

**Affiliations:** 1 Department of Oncology/Hematology, Kyungpook National University Medical Center, Kyungpook National University School of Medicine, Kyungpook National University Cancer Research Institute, 807 Hogukno, buk-gu, Daegu 41404, Korea; bwkang@knu.ac.kr; 2 Department of Surgery, Kyungpook National University Hospital, Kyungpook National University School of Medicine, Daegu 41494, Korea; kok007@hanmail.net (O.-K.K.); hychung@knu.ac.kr (H.Y.C.); wyu@knu.ac.kr (W.Y.)

**Keywords:** gastric cancer, taxanes, paclitaxel, docetaxel, chemotherapy

## Abstract

Although rapid advances in treatment options have improved the prognosis of advanced gastric cancer (AGC), it remains a major public health problem and the second leading cause of cancer-related deaths in the world. Taxanes (paclitaxel and docetaxel) are microtubule stabilizing agents that inhibit the process of cell division, and have shown antitumor activity in the treatment of AGC as a single or combination chemotherapy. Accordingly, this review focuses on the efficacy and tolerability of taxanes in the first- or second-line chemotherapy setting for AGC.

## 1. Background

Although rapid advances in treatment options have improved the prognosis of advanced gastric cancer (AGC), it remains a major public health problem and the second leading cause of cancer-related deaths in the world [1,2]. For metastatic or recurrent gastric cancer, one of the most important treatment modality is systemic chemotherapy, yet the optimum standard chemotherapy regimen for AGC remains debatable, and most responses to chemotherapy are partial and short-term.

Cisplatin is a small-molecule platinum compound that forms intrastrand cross-links, which activate the apoptotic pathway, resulting in cell death [3]. Cisplatin has had a significant impact on the treatment of AGC. A doublet combination regimen of either cisplatin-based or 5-fluorouracil (5FU)-based chemotherapy has been regarded as standard treatment for AGC [4]. Previous studies have shown that a cisplatin-based combination was marginally superior to other combinations, while the survival improvement is still disappointing, with a high rate of toxicity [5]. The significant toxicity of cisplatin, which frequently produces nausea, vomiting, neurotoxicity, and nephrotoxicity, can also affect the treatment outcomes and quality of life in patients with advanced stage AGC.

Meanwhile, the taxanes (paclitaxel or docetaxel) disrupt the microtubule function and inhibit the process of cell division, and have shown encouraging activity as a single or combination chemotherapy for the treatment of AGC [6,7]. Accordingly, this review focuses on the efficacy and tolerability of taxanes in a first- or second-line chemotherapy setting for AGC.

## 2. Overview of First- and Second-Line Chemotherapy for AGC

Platinum-based doublet chemotherapy has been universally accepted as the standard first-line treatment for AGC in many countries [8]. S-1 is an oral anticancer agent that is a combination of gimeracil, oteracil, and tegafur. Tegafur is a prodrug of 5FU, while gimeracil inhibits the degradation of 5FU by blocking a dehydrogenase enzyme and oteracil reduces the production of 5FU in the gut (Table 1) [9]. In a randomized phase III trial (SPIRITS trial) that compared S-1 plus cisplatin with S-1 alone in 298 patients with AGC, treatment with S-1 plus cisplatin was associated with improved median progression-free survival (PFS) (6.0 *vs.* 4.0 months) and overall survival (OS) (13.0 *vs.* 11.0 months), respectively. Plus, the response (54% *vs.* 31%) was also higher [10]. Therefore, based on this trial, an S-1 plus cisplatin combination regimen has been established as the standard first-line treatment for AGC in Japan. Meanwhile, another large randomized phase III trial (REAL-2 trial), conducted in Western countries, compared capecitabine with 5FU and oxaliplatin with cisplatin in 1003 patients with AGC. The results from this trial indicated that capecitabine is non-inferior to infused 5FU and oxaliplatin is non-inferior to cisplatin, respectively [11]. Plus, the ML17032 phase III trial also evaluated the combination of capecitabine and cisplatin *vs.* the combination of 5FU and cisplatin in AGC patients [12]. Here, the median PFS (5.6 *vs.* 5.0 months) and OS (10.5 *vs.* 9.3 months) were comparable in both groups. Consequently, an oral 5FU (capeciatbine or S-1) and platinum-based combination is now invariably used worldwide as the first-line choice for patients with AGC. Moreover, a more recent phase III trial showed that the addition of trastuzumab to a cisplatin–based chemotherapy significantly improved the survival of patients with HER2-positive AGC [13]. Thus, while the frequency of HER2 overexpression is low (10%–20%), a cytotoxic chemotherapeutic regimen with trastuzumab is regarded as the standard first-line treatment for HER2-positive AGC.

In clinical practice, various new chemotherapeutic agents, including oral 5FU, docetaxel, paclitaxel, and irinotecan, have been tested as a second-line chemotherapy. In particular, paclitaxel, docetaxel, and irinotecan have been identified promising drugs whether in combination regimens or as single agents. According to the results of several recent studies, the patient response rates ranged from approximately 10% to 20%, and the PFS ranged from 2.5 to 4.0 months [14]. Interestingly, the survival period following the failure of first-line chemotherapy has been shown to be longer in Asia than in Western countries, possibly due to the higher proportion of patients who receive subsequent chemotherapy in Asia and differences in the patient characteristics [15].

## 3. Clinical Efficacy of Taxanes in the Treatment of AGC

Taxanes are diterpenes produced by plants in the genus *Taxus* (yews). As their name suggests, taxanes were first derived from natural sources, yet now are synthesized artificially (Table 1) [16]. Docetaxel and paclitaxel belong to the taxane family as their chemical structures contain a common three-phenol ring, and both have therapeutic indications for solid tumor malignancies, including ovarian cancer, breast cancer, lung cancer, and gastric cancer [17]. Moreover, recent clinical studies have investigated the activity of several new drugs, such as nanoparticle albumin-bound paclitaxel (nab-paclitaxel) and cabazitaxel in gastric cancer.

### 3.1. Docetaxel

Based on several phase II studies, docetaxel has been identified as improving the outcomes for AGC. Thus, in order to evaluate whether the addition of docetaxel improves survival when used with a doublet including cisplatin and 5FU (DCF), Van Cutsem *et al.* conducted a single international randomized phase III trial (V-325 trial) among 445 patients with AGC. The addition of docetaxel achieved significant improvements in time to progression (TTP) (5.6 *vs.* 3.7 months) and OS survival (9.2 *vs.* 8.6 months) [18]. However, serious adverse effects of treatment were also higher among patients receiving DCF. They mainly included neutropenia (grade 3–4, 82% *vs.* 57%), febrile neutropenia (29% *vs.* 12%) and diarrhea (grade 3–4, 19% *vs.* 8%). Plus, while the trial only showed a limited quantitative benefit for survival, quality of life and clinical benefit were found to be favorable for DCF and were not compromised by the higher toxicity that accompanies the more active triplet regimen [19,20]. Therefore, these findings suggest that DCF regimen can also be used as a first-line treatment for patients with AGC who are able to tolerate this combination. Different combinations with capecitabine, S-1, cisplatin, oxaliplatin, and 5FU have also been examined as a first-line setting in phase II studies, with interesting results (Table 2) [21,22,23,24]. In particular, recent triple combination treatment with docetaxel, S-1, and cisplatin was generally safe and effective in patients with AGC. This study indicated that the triple combination provided a high response rate of 87.1% and had an acceptable toxicity profile [25].

Second-line chemotherapy has been tested in several randomized studies (Table 3). The recent trial conducted by Korean investigators included 202 patients who received either chemotherapy (irinotecan 150 mg/m^2^ every 2 weeks or docetaxel 60 mg/m^2^ every 3 weeks) or best supportive care [26]. All patients received at least one prior therapy were randomized in a 2:1 fashion and the choice of chemotherapy was left to the discretion of the attending physician. The study reported that the chemotherapy group was associated with a significant improvement in the median OS (5.3 months) *vs.* best supportive care (3.8 months) (hazard ratio = 0.657, *p* = 0.007). No difference was noted in the treatment effect of docetaxel or irinotecan (*p* = 0.116). A further multivariate analysis revealed that the performance status, prior chemotherapy, and chemotherapy-free interval were all prognostic factors, which is consistent with the prognostic model provided in a retrospective study by Hasegawa *et al.* [27]. Another phase III trial (COUGAR-02) also showed that docetaxel (75 mg/m^2^ every 3 weeks) significantly prolonged the median OS (5.2 months *vs.* 3.6 months, hazard ratio = 0.67, *p* = 0.01) [28]. While docetaxel chemotherapy was also associated with a higher incidence of grade 3–4 neutropenia and febrile neutropenia, the symptom scores for pain were significantly better. Overall, it is noteworthy that these trials showed comparable clinical benefits in different patient populations as regards the survival outcomes and tolerability, except that the chemotherapy in the Korean trial was continued until disease progression. A recent meta-analysis by Kim *et al.* evaluated the efficacy of cytotoxic chemotherapy (docetaxel or irinotecan) *vs.* best supportive care as a second-line treatment in patients with AGC [29] . Based on 410 patients in 578 studies, where three trials used pooled estimates for OS, the final hazard ratio for OS of 0.64 showed a significant difference between chemotherapy and best supportive care. Therefore, these findings suggest that both drugs (taxanes and irinotecan) are active as second-line chemotherapy in the case of AGC.

### 3.2. Paclitaxel

Several studies have already reported a benefit of first-line paclitaxel chemotherapy for AGC and responses rates were in the range of 17% to 28% [7]. Paclitaxel scheduled every 3 weeks as a first-line treatment for AGC patients has shown a response rate of 17% and the median survival was 8 months. A dose of 200 mg/m^2^ was also generally well tolerated [35]. Meanwhile, Emi *et al.* have reported that a weekly dose of paclitaxel at 80 mg/m^2^ was active and well tolerated. The overall response rate of 17.6% was reported with the median survival of 7.3 months and manageable toxicity profiles [36]. The combination of paclitaxel and S-1 was also tested in a randomized phase II trial and provided an acceptable safety profile and modest activity with a response rate of 52.3% [32]. Therefore, based on these trials, a paclitaxel-containing regimen can be considered as a practicable alternative to a platinum combination.

Although many drugs have already been tested and shown to be active [37], the most appropriate second-line chemotherapy regimen for AGC remains unclear. In a Japanese trial (WJOG4007), 223 patients without severe peritoneal metastasis following the failure of 5FU plus platinum combination chemotherapy were randomly assigned to irinotecan monotherapy (150 mg/m^2^ days 1 and 15 every 4 weeks) *vs.* weekly paclitaxel (80 mg/m^2^ days 1, 8, and 15 every 4 weeks) [33]. Neither regimen was found to be superior in terms of efficacy or tolerability. Meanwhile, the REGARD phase III trial evaluated ramucirumab, a fully IgG1 anti-vascular endothelial growth factor receptor-2 monoclonal antibody, *vs.* a placebo in patients with disease progression following first-line chemotherapy [38]. In this trial, ramucirumab demonstrated a significantly improved median OS when compared to the placebo group (5.2 months *vs.* 3.8 months, hazard ratio = 0.776, *p* = 0.047). Plus, the subsequent RAINBOW phase III trial, which compared paclitaxel with or without ramucirumab as a second-line chemotherapy, also showed a significantly prolonged OS for the combination arm with ramucirumab [34]. Thus, the success of ramucirumab and paclitaxel in a second-line setting has prompted ongoing clinical investigations in a first-line setting. Moreover, since paclitaxel is known to regulate multiple actions in the immune system by exerting different effects on immune cells, further intensive research is needed in combination with tumor microenvironment modulating agents, such as ramucirumab, to reveal the potential role of paclitaxel with target agents [39].

### 3.3. Nanoparticle Albumin-Bound Paclitaxel

Nanoparticle albumin-bound paclitaxel (Nab-paclitaxel) is a novel, solvent polyoxyethylated castor oil (Cremophor)-free, and biologically interactive form of paclitaxel [17]. Nab-paclitaxel utilizes the natural properties of albumin to reversibly bind paclitaxel, transport it across the endothelial cell and concentrate it in areas of tumor. Nab-paclitaxel allows the administration of paclitaxel without the use of lipid-based solvents, shorter infusion schedules, and no requirement of premedication for solvent-based hypersensitivity reactions. Moreover, nab-paclitaxel documented increased paclitaxel transport across endothelial cells and greater antitumor activity when compared with standard paclitaxel in a preclinical study [40]. In several phase III trials, nab-paclitaxel showed an improved response rate compared to paclitaxel in patients with breast or non-small cell lung cancer [41,42]. More recently, the efficacy and safety of nab-paclitaxel was investigated in 56 patients with unresectable or recurrent gastric cancer who experienced progression despite fluoropyrimidine-containing treatment [43]. Nab-paclitaxel was given intravenously at 260 mg/m^2^ on day 1 of each 21-day cycle without anti-allergic premedication until disease progression. With an overall response rate of 27.8% and disease control rate of 59.3%, the median PFS and OS were 2.9 months (95% CI, 2.4–3.6) and 9.2 months (95% CI, 6.9–11.4), respectively. Significant hypersensitivity or anaphylactic reactions were not observed. To evaluate the clinical benefit of nab-paclitaxel as a second-line chemotherapy for AGC, a randomized phase III trial is now ongoing in Japan.

### 3.4. Cabazitaxel

Cabazitaxel is a semi-synthetic microtubule inhibitor that is known for its ability to inhibit cancer cell growth in both docetaxel-sensitive and docetaxel-resistant cell lines [30]. Cabazitaxel, a novel second-generation taxane, has also exhibited increased potency when compared with docetaxel in taxane-resistant tumor models and demonstrated antitumor activity in preclinical models of gastric cancer [44]. In a gastric cancer phase I study of cabazitaxel (15–25 mg/m^2^, 1 h infusion every 3 weeks), 15 mg/m^2^ was confirmed as the maximum tolerated dose [45]. At a dose of 20–25 mg/m^2^, prolonged neutropenia and febrile neutropenia were frequently seen and one patient had a partial response at 15 mg/m^2^. Such data indicated that high rate of neutropenia with cabazitaxel could result in increased myelotoxicity in Asian patients. Thus, ongoing two phase II trials will clarify the role of cabazitaxel in patients with refractory gastric cancer (NCT01757171, NCT01956149).

## 4. Adverse Effects of Taxanes

The toxicity of such agents includes bone marrow suppression, alopecia, and hypersensitivity reactions [46]. While both docetaxel and paclitaxel can cause neurotoxicity and myalgia, this is a greater clinical concern with paclitaxel. Docetaxel has also been associated with the development of significant fluid retention, the incidence and severity of which can be limited by prophylactic treatment with corticosteroids both before and after each treatment. If patients are monitored closely, taxanes have a favorable side-effect profile, and it is currently uncommon for treatment to be discontinued because of the development of excessive toxicity. To lower the inherent toxicity and side effects of these drugs, various taxane delivery systems have already been developed, including nanoparticles, liposomes, micelles, bioconjugates, and dendrimers [47,48]. These nanocarriers offer a high degree of encapsulation and cellular uptake, escape elimination by P-glycoprotein mediated efflux, and can be used for targeted drug delivery. Thus, various taxane nanocarriers are currently in different stages of clinical trials and a few have already been commercialized, such as nab-paclitaxel. For scheduling, docetaxel and paclitaxel have been studied for a 3-weekly *vs.* weekly schedule, resulting in similar efficacy and tolerability with a weekly treatment [49]. Thus, the choice of schedule should be balanced between the convenience of fewer clinic visits and the tolerability of treatment when selecting the optimal treatment of taxanes.

## 5. Conclusions

A doublet combination regimen of either cisplatin-based or 5FU-based chemotherapy has been regarded as standard treatment for AGC. Notwithstanding, taxanes are clearly merging as an important treatment for patients with AGC. In the current review, taxanes have been shown to be effective and tolerable as first-line and second-line chemotherapeutic agents. Paclitaxel combined with ramucirumab have also been identified as notably useful for second-line treatment. Accordingly, taxanes can be a reasonable therapeutic choice when treating AGC. Additional trials are needed to refine the benefits of taxanes, and further evidence is needed to make improvements in the management of AGC.

## Figures and Tables

**Table 1:** Molecular structures of taxanes and S-1 components.

Name	Molecular Structure	Reference
Docetaxel	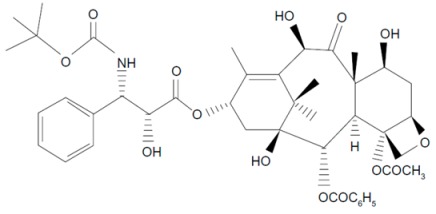	[16,30]
Paclitaxel Nanoparticle albumin-bound paclitaxel	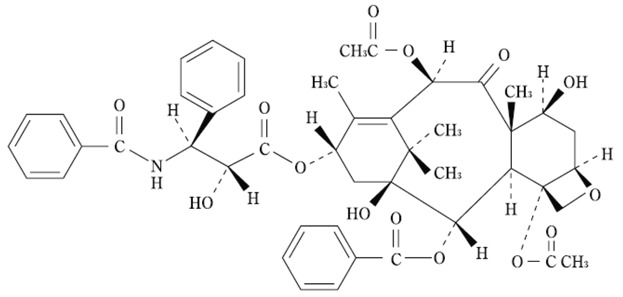	[7,31]
Cabazitaxel	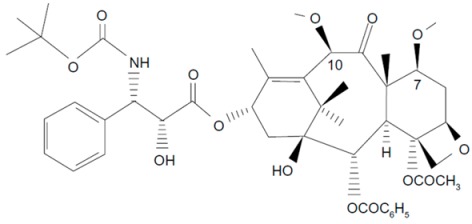	[17,30]
S-1	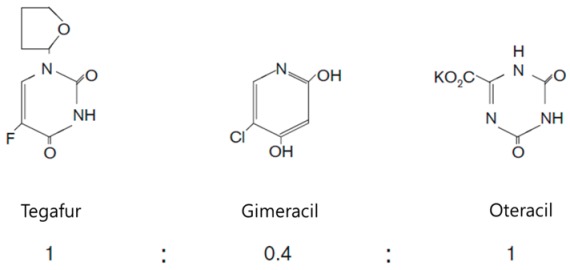	[9]

**Table 2:** Selected phase II and III trials of first-line taxane-based chemotherapy for patients with advanced gastric cancer.

Author	Year	Phase	Tx	Dose (mg/m^2^ per day)	Cycle	Patients (*n*)	RR (%)	Median TTP/TTF/PFS (mo)	Median OS (mo)	*p*-Value for OS
Van Cutsem *et al.* [18]	2006	III	DCF	(5FU:750, C:75, D:75)	(D1–5, D1, and D1, 3 weeks)	221	37	5.6	9.2	0.0201
			CF	(F:1000, C:100)	(D1–5 and D1)	224	25	3.7	8.6	
Sato *et al.* [25]	2010	II	DCS	(D:60, C:60, S:80)	(D8, D8 and D1–14, 3 weeks)	34	87.1	226 (days)	687 (days)	-
Van Cutsem *et al.* [24]	2015	II (random)	DOx	(D:75, Ox:130)	(D1, 3 weeks)	64	23.1	4.5	8.97	-
			DOxF	(D:50, Ox:85, F:2400)	(D1, D1, and D1–2, 3 weeks)	79	46.6	7.66	14.59	
			DOxX	(D1:50, Ox:100, X:1250)	(D1, D1 and D1–14, 3 weeks)	63	25.6	5.55	11.30	
Kim *et al.* [22]	2014	II (random)	DC	(D:35, C:60)	(D1, 8 and D1, 3 weeks)	38	37	4.9	9.7	0.581
			DOx	(D:35, Ox:120)	(D1, 8 and D1, 3 weeks)	38	41	4.4	12.3	
Jeung *et al.* [21]	2011	II (random)	DS	(D:35, S:70)	(D1, 8 and D1–14, 3 weeks)	39	46	7.3	16.0	0.019
			DC	(D:35, C:35)	(D1, 8 and D1, 8, 3 weeks)	41	24	4.8	8.2	
Park *et al.* [23]	2006	II (random)	PF	(P:175, F:500)	(D1 and D1–5, 3 weeks)	38	42	3.6	9.9	-
			DF	(D:75, F:500)	(D1 and D1–5, 3 weeks)	39	33	4.2	9.3	
Mochiki *et al.* [32]	2012	II (random)	SP	(S:80, P:60)	(D1–14 and D1, 8, 15, 4 weeks)	42	52.3	9.0	16.0	0.084
			SC	(S:80, C:60)	(D1–21 and D8, 5 weeks)	41	48.7	6.0	17.0	

Abbreviations: Tx: Treatment; TTP: Time to progression; TTF: Time to treatment failure; PFS: Progression-free survival; OS: Overall survival; F: 5FU; C: Cisplatin; S: S-1; D: Docetaxel; X: Capecitabine; P: Paclitaxel.

**Table 3:** Phase III trials of second-line taxane-based chemotherapy for patients with advanced gastric cancer.

Author	Indication	Study Name	Agents	Number	Response Rate (%)	Overall Survival (Months)	Hazard Ratio & *p*-Value
Hironaka *et al.* [33]	Second-line	WJOG4007, Phase III	Irinotecan 150 mg/m^2^ every 2 weeks	111	-	8.4	1.13 (0.86–1.49) *p* = 0.38
			Paclitaxel 80 mg/m^2^ days 1, 8, 15 every 4 weeks	108	-	9.5	
Kang *et al.* [26]	Second or third-line	Korean, Phase III	Docetaxel 60 mg/m^2^ every 3 weeks	133	11	5.3	0.657 (0.485–0.891) *p* = 0.007
			Irinotecan 150 mg/m^2^ every 2 weeks	-	8	-	
			Best supportive care ^†^	69	-	3.8	
Ford *et al.* [28]	Second-line	COUGAR-02, Phase III	Docetaxel 75 mg/m^2^ every 3 weeks	84	7	5.2	0.67 (0.49–0.92) *p* = 0.01
			Best supportive care ^†^	84	-	3.6	
Wilke *et al.* [34]	Second-line	RAINBOW, Phase III	Ramucirumab 8 mg/kg days 1 and 15, and paclitaxel 80 mg/m^2^ days 1, 8, and 15 every 4 weeks	330	28	9.6	0.807 (0.678–0.962) *p* = 0.017
			Paclitaxel 80 mg/m^2^ days 1, 8, and 15 every 4 weeks	335	16	7.4	

^†^ Active symptom control.
